# Quantitative and Qualitative MRI Assessment of Perivascular Spaces in Parkinson’s Disease Patients †

**DOI:** 10.3390/medicina62040613

**Published:** 2026-03-24

**Authors:** Evelina Stagisa, Arturs Silovs, Gvido Karlis Skuburs, Nauris Zdanovskis, Aleksejs Sevcenko, Janis Mednieks, Edgars Naudins, Santa Bartusevica, Solvita Umbrasko, Liga Zarina, Laura Zelge, Agnese Anna Pastare, Jelena Smilga, Jurgis Skilters, Baingio Pinna, Ardis Platkajis

**Affiliations:** 1 Department of Radiology, Riga East University Hospital, Hipokrata Street 2, LV-1038 Riga, Latvia; arturs.silovs@rsu.lv (A.S.);; 2 Department of Radiology, Riga Stradins University, Dzirciema Street 16, LV-1007 Riga, Latvia; 3 Latvian Maritime Medicine Centre, Patversmes Street 29, LV-1005 Riga, Latvia; 4 Department of Neurology, Riga Stradins University, Dzirciema Street 16, LV-1007 Riga, Latvia; 5 Laboratory for Perceptual and Cognitive Systems, University of Latvia, Raiņa Bulvāris 19, LV-1586 Riga, Latvia; 6 Department of Biomedical Sciences, University of Sassari, 07100 Sassari, Italy

**Keywords:** Parkinson’s disease, enlarged perivascular spaces, cognitive impairment, MRI, basal ganglia, centrum semiovale, glymphatic system, neurodegeneration

## Abstract

*Background and Objectives*: Enlarged perivascular spaces (ePVS) demonstrated by MRI have recently been associated with cerebral small vessel disease and glymphatic dysfunction, implicated in Parkinson’s disease (PD) pathophysiology. This study aimed to quantify the burden of ePVS in PD patients versus healthy controls and to examine associations with cognitive performance. *Materials and Methods*: A total of 51 participants underwent 3T MRI, including a T2-weighted sequence. Twenty-one patients with Parkinson’s disease and 21 age-matched healthy controls were included in the final analysis. The ePVS burden was assessed quantitatively by counting visible PVS in the basal ganglia and centrum semiovale, and qualitatively using Potter and Heier rating scales. Cognitive function was measured with the Montreal Cognitive Assessment (MoCA). Statistical analyses used Mann–Whitney U tests and Spearman correlations. *Results*: PD patients had significantly higher total PVS counts in the basal ganglia (84.8 vs. 48.0; *p* < 0.001) and centrum semiovale (290.6 vs. 143.9; *p* < 0.001). Potter scale ratings were higher in PD across regions (*p* ≤ 0.025). Largest per-slice PVS counts negatively correlated with MoCA scores in right basal ganglia (ρ = −0.362, *p* = 0.012) and bilateral centrum semiovale (right: ρ = −0.421, *p* = 0.003; left: ρ = −0.431, *p* = 0.002). Heier scale differences were significant only in the right centrum semiovale (*p* = 0.023). PVS diameters were larger in PD only in the centrum semiovale (right: *p* = 0.010; left: *p* = 0.040). *Conclusions*: In this cohort, increased ePVS burden in the basal ganglia and centrum semiovale was associated with cognitive impairment in PD patients. Qualitative and quantitative PVS assessment, notably the largest-per-slice counts, may serve as a sensitive, non-invasive imaging biomarker for neurodegeneration and cognitive decline in PD. Larger group studies and longitudinal data are needed to assess their prognostic value in the long term, as well as the development of automatic quantification applications for better reproducibility.

## 1. Introduction

### 1.1. Perivascular Spaces in Neurodegeneration

The perivascular space (PVS), also known as the Virchow–Robin space, is a fluid-filled cavity that surrounds small blood vessels in the brain and is typically less than 2 mm in diameter [1]. It facilitates the exchange of fluids between cerebrospinal fluid and brain parenchyma, functions analogously to lymphatic vessels, and plays a vital role in waste clearance and neurovascular homeostasis [2].

The enlargement of PVS is thought to be the result of several interacting mechanisms. Age-related stiffening of small arteries reduces their elasticity, altering fluid clearance along the perivascular pathways and leading to dilation [3]. The accumulation of misfolded proteins, such as α-synuclein, in neurodegenerative conditions can obstruct drainage routes, promoting fluid retention within PVS [4]. In addition, brain atrophy associated with ageing can create additional space around vessels, facilitating PVS expansion [5]. Disruption of the blood-brain barrier increases vascular permeability, allowing fluid and protein leakage into PVS and further contributing to their enlargement [4,6].

Under normal physiological conditions, PVS are not usually detectable on MRI. However, glymphatic dysfunction and age-related changes can lead to their enlargement, allowing visualisation [7]. If PVS are visible on MRI and are larger than 2 mm, they are generally classified as enlarged. Enlarged perivascular spaces (ePVS) are more frequently found in the centrum semiovale and basal ganglia, with lesser occurrences in the midbrain and hippocampus [4]. Recommended sequences for PVS visualization are T1-weighted imaging (T1WI), T2-weighted imaging (T2WI), fluid attenuated inversion recovery (FLAIR), diffusion-weighted imaging (DWI), and apparent diffusion coefficient (ADC) mapping [8]. However, they are best visualised on T2WI, where they appear as hyperintense structures, allowing a clear distinction from surrounding brain tissue [9]. These PVS typically appear as clearly defined oval, round, or tubular structures with smooth margins, often seen in bilateral clusters of varying sizes, although mild asymmetry in their distribution is common [10].

To evaluate the severity and extent of PVS, several visual rating scales have been developed and are commonly used in neuroimaging studies. Among these, the Potter scoring system is the most widely applied [8]. These systems differ in whether they evaluate the number, size, or anatomical distribution of PVS and provide complementary approaches to characterise the PVS burden.

Although PVS was once considered a normal anatomical characteristic, they are now increasingly recognised as a marker of small vessel disease of the brain [11]. Moreover, PVS are considered potential indicators for various neurological disorders, including Parkinson’s disease [12].

### 1.2. Perivascular Spaces as Cognitive Biomarkers in Parkinson’s Disease

Parkinson’s disease (PD) is a neurodegenerative disorder characterised by a wide spectrum of clinical manifestations. It is a chronic condition that progressively worsens over time, with symptoms gradually intensifying as the disease progresses [13]. Its motor symptoms typically include resting tremor, bradykinesia, muscular rigidity, and gait disturbances, while non-motor symptoms often include hyposmia, sleep disturbances, and mood or affective disorders [14,15]. The aetiology of PD is considered multifactorial, involving both genetic predisposition and environmental influences [16].

The pathophysiology of PD involves the progressive degeneration of dopamine-producing neurons in the substantia nigra of the midbrain [17]. A key pathological hallmark is the misfolding and aggregation of α-synuclein in Lewy bodies, which affects neuronal function and promotes cell death [18,19]. Mitochondrial dysfunction, oxidative stress, impaired protein clearance, and chronic neuroinflammation further contribute to dopaminergic loss, leading to motor and non-motor symptoms [20].

Recent research highlights that dysfunction of the glymphatic system, which facilitates the clearance of interstitial waste, including misfolded proteins such as α-synuclein, exacerbates the progression of PD by promoting protein aggregation and neuroinflammation [16]. Impairments in this clearance pathway lead to ePVS, visible on MRI, thereby linking structural brain changes to molecular pathology in PD [21]. In addition, genetic mutations affecting the lysosomal and autophagy–lysosomal pathways further compromise protein degradation, contributing to neurotoxicity and loss of dopaminergic neurons [16]. These mechanisms create a feedback loop where impaired protein clearance and vascular dysfunction accelerate neurodegeneration and worsen both motor and cognitive symptoms, emphasising the multifactorial nature of PD pathophysiology [20]. Clinically, the burden of ePVS in specific brain regions has been associated with the severity of symptoms in PD. In the basal ganglia, enlarged and more numerous PVS correlate with increased severity of motor symptoms and cognitive decline, while abnormal PVS changes in white matter have been associated with gait freezing [22].

Mild cognitive impairment occurs frequently in PD, including during its early stages, and can increase the risk of progression to dementia [23]. The Montreal Cognitive Assessment (MoCA) is widely used as a comprehensive cognitive screening tool in this context [24]. Moreover, ePVS observed in the basal ganglia and white matter has been correlated with disease severity, cognitive impairment, and poorer performance on the MoCA in patients with PD [25], indicating that ePVS in the basal ganglia and centrum semiovale serve as MRI-visible markers corresponding to underlying pathological mechanisms that contribute to the progression of PD [26].

This study aimed to compare ePVS burden between PD patients and healthy controls using quantitative counts and qualitative rating scales, and to investigate associations between ePVS measures and MoCA cognitive performance.

## 2. Materials and Methods

### 2.1. Participants

This study included 51 participants. One participant was excluded due to an incomplete MRI examination. To minimise age-related confounding, age outliers were excluded using the 1.5 × IQR criterion. Subsequently, Parkinson’s disease patients were matched 1:1 with healthy controls using age-matched nearest-neighbour matching. All participants provided written informed consent, and this study was approved by the Institutional Ethics Committee of Paul Stradins University. Inclusion criteria for PD patients included a clinical diagnosis established according to the Movement Disorder Society (MDS) clinical diagnostic criteria for Parkinson’s disease [27]. This diagnosis was made by a multidisciplinary team of neurologists, with atypical Parkinsonian syndromes systematically excluded. Additional criteria comprised a disease duration not exceeding 10 years and the ability to undergo MRI scanning. MRI examinations were conducted without adjustment for dopaminergic medication status.

### 2.2. MRI Acquisition

All MRI scans were acquired on a 3 Tesla scanner (GE HealthCare, Chicago, IL, USA) using a standardised imaging protocol, which included a T2-weighted fast spin-echo (FSE) sequence. The key imaging parameters were as follows: repetition time (TR) = 4653 ms, echo time (TE) = 120 ms, slice thickness = 2.0 mm, spacing = 0.5 mm, field of view (FOV) = 24 cm × 21.6 cm, voxel size = 0.8 × 0.9 × 2.0 mm^3^. Imaging covered the basal ganglia (BG), centrum semiovale (CS), and other relevant brain regions.

### 2.3. Quantification of Perivascular Spaces

PVS were assessed using quantitative methods. Three neuroradiologists independently assessed and marked visible enlarged perivascular spaces bilaterally in the basal ganglia and centrum semiovale while blinded to the clinical diagnosis. Subsequently, disagreements were resolved during a consensus review session, and consensus ratings were used for all quantitative analyses. As only consensus-level data were retained for the final dataset, formal inter-rater reliability statistics (e.g., intraclass correlation coefficients) could not be calculated.

Additionally, the largest number of ePVS observed on any single MRI slice was recorded separately for each hemisphere. Both the total count and largest-per-slice counts from the right and left hemispheres were included in subsequent analyses. Several visual rating scales have been proposed for the assessment of perivascular spaces. A comparative overview of commonly used scoring systems is presented in Table 1.

Based on the characteristics and clinical applicability of these systems, two visual rating scales were selected for the present study. The Potter scale [28] was used to grade PVS severity on a 0–4 grading scale based on the number within the basal ganglia and centrum semiovale:

- grade 0 indicates no visible PVS,
- grade 1 corresponds to less than 10,
- grade 2 to 11–20,
- grade 3 to 21–40, and
- grade 4 to more than 40 PVS.

The scoring was applied independently to both hemispheres. However, a simplified 0–1 scale is used in the midbrain to reflect PVS absence or presence [8]. This region was not included in the present analysis.

In addition, the Heier scale [29] was used to evaluate the maximal diameter of PVS, providing a complementary morphological measure. PVS were classified into three grades on the basis of their diameter:

- grade 1 (<2 mm),
- grade 2 (2–3 mm),
- grade 3 (>3 mm).

This approach allowed for the simultaneous evaluation of both the extent and morphological characteristics of PVS.

### 2.4. Cognitive Assessment

The MoCA was administered to assess cognitive function. The MoCA is a 30-point screening tool that evaluates multiple cognitive domains, including memory, attention, executive functions, language, visuoconstructional skills, conceptual thinking, calculations, and orientation. A score of 26 or higher is generally considered indicative of normal cognition [33].

### 2.5. Statistical Analysis

Group differences in PVS burden were evaluated using the Mann–Whitney U test due to non-normal data distribution, as determined by the Shapiro–Wilk tests. The association between PVS burden and cognitive performance was assessed using the Spearman rank correlation coefficient. Fisher’s exact test was applied for categorical comparisons of PVS burden. Statistical significance was set at *p* < 0.05. Effect sizes, reported as rank-biserial correlation (r_rβ_), were calculated for quantitative variables. All analyses were conducted using IBM SPSS version 29.0.

## 3. Results

### 3.1. Demographic Characteristics

After exclusion of age outliers and 1:1 age matching, PD patients (*n* = 21) and controls (*n* = 21) showed comparable age distributions (median age 64 years in both groups).

Patients exhibited lower cognitive performance compared to controls. MoCA scores deviated from normality (Shapiro–Wilk *p* < 0.001), and group differences were, therefore, assessed using the Mann–Whitney U test, revealing significantly lower cognitive performance in PD patients compared with controls (*p* < 0.001). No significant age or gender differences were observed between the groups after matching.

Demographic characteristics are summarised in Table 2.

### 3.2. Quantitative PVS Measures

Patients exhibited a significantly higher PVS burden compared to controls in all regions evaluated. The total PVS counts of the basal ganglia were 84.8 ± 41.3 in patients and 48.0 ± 24.5 in controls (*p* < 0.001). Similarly, centrum semiovale PVS burden was markedly elevated in patients, 290.6 ± 116.3, compared to 143.9 ± 80.5 in controls (*p* < 0.001).

### 3.3. Visual Rating Scales

Potter scale ratings confirmed a significantly greater PVS burden in PD patients, compared to the controls in the basal ganglia and centrum semiovale bilaterally, as demonstrated in Table 3.

For categorical neuroimaging variables, Fisher’s exact test was used due to expected cell counts < 5. Fisher’s exact tests revealed statistically significant group differences in PVS burden categories, as defined by the Potter scale, in basal ganglia right (*p* < 0.001), basal ganglia left (*p* = 0.025), centrum semiovale right (*p* = 0.001), and centrum semiovale left (*p* = 0.002).

For the Heier scale, which categorises PVS by maximum diameter, no significant differences were found between patients and controls in the basal ganglia in either the right (*p* = 0.306) or left hemisphere (*p* = 0.809). In the centrum semiovale, however, patients had significantly higher Heier categories on the right side compared to controls (*p* = 0.023), while the left side showed no significant difference (*p* = 0.548). Effect sizes (Phi = 0.357 for the MoCA group) supported meaningful associations between cognitive status and PVS measures.

### 3.4. Largest-per-Slice PVS Counts

**Figure 1:**
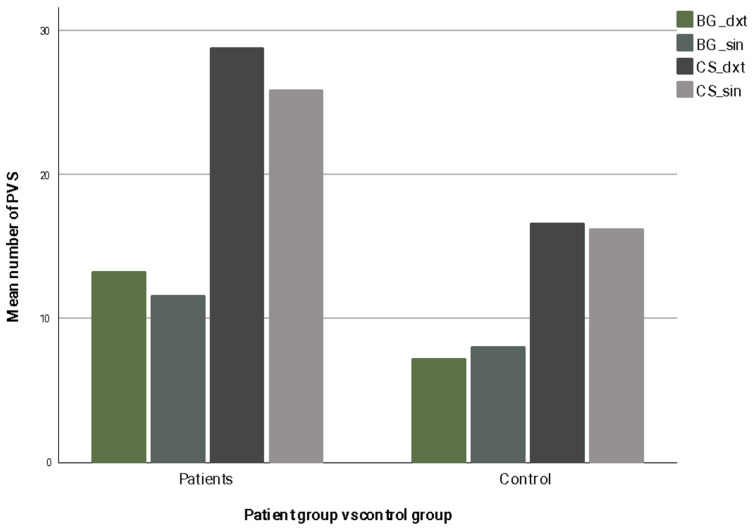
Largest PVS count per slice across four brain regions in patients and controls. Across all examined brain regions, patients exhibited a higher largest-per-slice PVS count compared with controls, with the most pronounced differences observed in the centrum semiovale. (“BG_dxt” right basal ganglia; “BG_sin” left basal ganglia; “CS_dxt” right centrum semiovale; “CS_sin” left centrum semiovale).

Patients exhibited significantly higher PVS counts per slice compared to controls in all regions of the brain examined. Specifically, in the right basal ganglia, the mean counts were 13.24 ± 6.77 for patients versus 7.21 ± 2.77 for controls (*p* < 0.001). Similarly, in the left basal ganglia, patients showed 11.57 ± 4.32 compared to 8.00 ± 2.75 in controls (*p* = 0.002). In the centrum semiovale, differences were even more pronounced: right hemisphere counts averaged 28.76 ± 9.87 in patients versus 16.59 ± 8.65 in controls (*p* < 0.001), and left hemisphere counts were 25.86 ± 8.03 compared to 16.21 ± 7.56 in controls (*p* < 0.001). *p*-values were calculated using the Mann–Whitney U test. To visualize the differences between patients and controls, the largest-per-slice PVS counts for each brain region are presented in Figure 1.

### 3.5. Perivascular Spaces Diameters

PVS diameters in the basal ganglia did not differ significantly between patients and controls. Specifically, the mean diameter in the right basal ganglia was 2.36 ± 0.60 mm in patients and 2.10 ± 0.56 mm in controls (*p* = 0.107), while in the left basal ganglia, mean diameters were 2.10 ± 0.55 mm and 2.19 ± 0.83 mm (*p* = 0.665). In the centrum semiovale, patients showed significantly larger PVS diameters compared to controls, with the right hemisphere measuring 2.13 ± 0.74 mm versus 1.70 ± 0.39 mm (*p* = 0.010) and the left hemisphere 1.89 ± 0.43 mm versus 1.69 ± 0.39 mm (*p* = 0.040). Group comparisons were conducted using Mann–Whitney U tests due to non-normal data distribution.

### 3.6. Associations with Cognitive Performance

**Figure 2:**
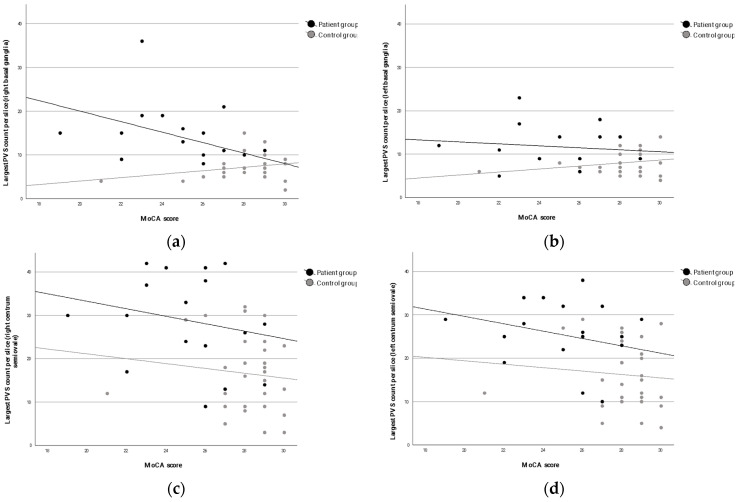
Scatterplots illustrating the associations between cognitive performance (MoCA) and the largest PVS count per slice across four brain regions: (**a**) right basal ganglia, (**b**) left basal ganglia, (**c**) right centrum semiovale, and (**d**) left centrum semiovale.

Spearman correlation analysis revealed that lower cognitive performance (MoCA score) was significantly associated with a higher largest-per-slice PVS count in three brain regions: right basal ganglia (ρ = −0.362, *p* = 0.012), right centrum semiovale (ρ = −0.421, *p* = 0.003), and left centrum semiovale (ρ = −0.431, *p* = 0.002). No significant association was found in the left basal ganglia (ρ = −0.144, *p* = 0.330). These findings indicate that the relationship between PVS burden and cognition is strongest in the centrum semiovale and shows partial lateralization to the right hemisphere. The associations between MoCA scores and the largest PVS count per slice in all brain regions are presented in Figure 2.

Overall, PD patients exhibited a significantly greater PVS burden across all metrics, particularly in the centrum semiovale, with partial lateralization to the right hemisphere. Sensitivity analyses, including the full dataset without exclusion of the age outlier, produced comparable results and did not alter the direction or statistical significance of the main findings (Supplementary Table S1).

## 4. Discussion

### 4.1. Main Findings

The present study demonstrates that patients with PD exhibit a significantly greater PVS burden in both the centrum semiovale (total count: 290.6 ± 116.3 vs. 143.9 ± 80.5; *p* < 0.001) and the basal ganglia (total count: 84.8 ± 41.3 vs. 48.0 ± 24.5; *p* < 0.001) compared with healthy controls. Group differences were most clearly demonstrated using quantitative PVS measures, particularly the largest-per-slice counts. Potter-scale ratings (categorically analysed) provided complementary evidence of an increase in PVS burden in the PD group, showing higher median grades for both basal ganglia (right: 2.0 vs. 1.0, *p* < 0.001; left: 2.0 vs. 1.0, *p* = 0.025) and centrum semiovale (right: 3.0 vs. 2.0, *p* < 0.001; left: 3.0 vs. 2.0, *p* = 0.001). Spearman correlation analyses further showed that a higher largest-per-slice PVS count was associated with lower MoCA scores, with the strongest relationships observed in the right basal ganglia (ρ = −0.362, *p* = 0.012) and bilateral centrum semiovale (right: ρ = −0.421, *p* = 0.003; left: ρ = −0.431, *p* = 0.002). The absence of a significant association in the left basal ganglia may indicate regional specificity or hemispheric lateralisation of PVS burden effects. Taken together, the findings indicate that increased PVS burden is associated with poorer cognitive performance in PD, highlighting its potential relevance as an imaging marker of disease-related cognitive decline. Nevertheless, the observed right-hemisphere predominance should be considered hypothesis-generating rather than confirmatory. Validation in larger independent cohorts and longitudinal studies will be necessary to determine whether hemispheric asymmetry represents a consistent feature of PVS involvement in Parkinson’s disease.

To our knowledge, this study is among the first to combine quantitative total PVS counts, largest-per-slice metrics, and qualitative visual grading to characterise regional PVS burden and its association with cognition in Parkinson’s disease. In particular, the largest-per-slice approach provides a clinically interpretable metric capturing focal PVS clustering that may not be reflected by total counts alone.

The largest-per-slice PVS metric may be particularly sensitive to disease-related microstructural changes, as it captures focal clustering of enlarged perivascular spaces rather than diffuse burden across the entire region of the brain. This approach may better reflect localised glymphatic dysfunction or regional impairment of perivascular clearance pathways, which are not fully represented by total PVS counts. Importantly, the largest-per-slice measurements are clinically intuitive and less dependent on exhaustive whole-brain counting, enhancing feasibility and reproducibility for routine clinical and research applications.

### 4.2. Interpretation and Comparison with the Literature

The findings of this study support previous reports that recognise PVS as MRI-visible markers of small vessel disease and glymphatic dysfunction of the brain, both implicated in the pathophysiology of PD [2,4,16]. Several studies have documented that enlarged PVS in the basal ganglia and centrum semiovale are more common in patients with PD and correlate with cognitive and motor impairment [22,25]. Donahue et al. [25] reported negative correlations between rostral, middle frontal PVS volume fraction and MoCA (r = −0.524, *p* < 0.001), as well as between basal ganglia PVS volume fraction and MoCA (r = −0.313, *p* = 0.029). Similarly, Shen et al. [26] reported that mean PVS number and volume in the right basal ganglia and midbrain were significantly higher in PD compared to healthy controls (mean PVS number: 12.4 ± 0.8 vs. 9.8 ± 0.7, *p* = 0.035; mean PVS volume: 41.5 ± 3.9 vs. 29.1 ± 2.7, *p* = 0.015), reinforcing that elevated PVS burden is a characteristic feature of PD. Despite methodological differences, particularly the use of volumetric measures in prior studies versus count-based largest-per-slice metrics in the present study, the overall findings converge in demonstrating a significant relationship between elevated PVS burden and cognitive impairment in PD. Consistent with these reports, the significant associations observed between MoCA scores and PVS counts in specific brain regions reinforce the role of PVS burden as a potential imaging biomarker for disease severity and progression [21,26].

Heier scale analysis, which classifies PVS by maximum diameter, revealed significant group differences only in the right centrum semiovale, consistent with previously reported regional variability of PVS involvement in PD. However, compared to the quantitatively largest-per-slice PVS counts, the Heier scale may provide less detailed information regarding PVS burden. Although stronger associations were predominantly observed in the right hemisphere, the mechanisms driving this lateralisation are poorly understood. PD is characterised by asymmetrical dopaminergic degeneration and compensatory processes [34], which, together with molecular and epigenetic hemispheric differences that influence neuronal vulnerability to α-synuclein pathology [35], can contribute to disease progression. In addition, asymmetric vascular and glymphatic dysfunction could lead to uneven accumulation of pathological factors and impaired clearance across the hemispheres [21,36].

These findings highlight the need for further investigation into how structural asymmetry might contribute to the variability in cognitive decline observed in PD. In general, this study strengthens the evidence that PVS quantification provides clinically meaningful information on microvascular and glymphatic alterations that affect functional outcomes in PD.

### 4.3. Strengths and Limitations

This study used complementary quantitative and qualitative methods, both of which confirmed a significantly higher burden of PVS in PD patients in multiple regions of the brain. Bilateral regional analysis of the centrum semiovale and basal ganglia enabled the detection of lateralization patterns, with significant right-hemisphere predominance. The MoCA was used to provide a consistent and reliable measure of cognitive performance, which supported correlations with PVS burden. However, several limitations should be acknowledged. While this study specifically examined MRI-visible perivascular spaces as an early PD biomarker, key structural changes linked to neurodegeneration were not evaluated, including cortical thickness, hippocampal and parahippocampal volumes, or basal ganglia dimensions. These established correlates of PD-related cognitive decline and dementia risk were omitted from the current analysis. Although age-related confounding was addressed through the exclusion of age outliers and subsequent age-matched sensitivity analyses, residual age effects cannot be completely excluded. The relatively small sample size may limit generalisability and statistical power, particularly for subgroup and lateralisation analyses. The cross-sectional design precludes causal inference on the temporal relationship between PVS burden and cognitive decline. MRI-based PVS assessment, while robust, may be influenced by image resolution and manual segmentation procedures, potentially affecting reproducibility between centres. Additionally, formal inter-rater reliability metrics could not be reported, as only consensus-based PVS measurements were retained; however, future studies should include systematic agreement analyses to further support reproducibility.

Finally, the observed hemispheric asymmetry in PVS burden and its association with cognition should be interpreted cautiously and warrant further investigation in larger longitudinal cohorts.

### 4.4. Clinical Implications

The demonstrated associations between region-specific PVS burden and cognitive impairment suggest that MRI-based PVS quantification may serve as a practical, non-invasive biomarker to identify PD patients at elevated risk of cognitive decline. Incorporating PVS assessment into routine clinical imaging could enhance early detection and risk stratification, particularly in individuals with marked involvement of the basal ganglia and centrum semiovale. As a complementary imaging marker, PVS quantification may support more individualised monitoring strategies and inform therapeutic approaches aimed at addressing glymphatic dysfunction and small-vessel pathology in PD.

### 4.5. Future Directions

Future longitudinal studies are needed to determine whether there are increases in PVS burden before or after cognitive decline in PD. Understanding this sequence is essential for evaluating PVS enlargement as a potential early biomarker. Automated PVS quantification methods should be further developed to improve reliability between studies. Future research should also examine whether improving glymphatic or vascular function through sleep-related interventions or vascular risk management can influence PVS burden or cognitive outcomes. Combining PVS measures with complementary structural and diffusion MRI techniques may help clarify underlying mechanisms and support personalised disease monitoring. In particular, diffusion-based assessment of white matter integrity may provide additional insight into visuospatial and attentional vulnerability in PD. Moreover, integrating PVS quantification with fluid biomarkers (e.g., CSF α-synuclein) and neurophysiological measures may deepen understanding of the pathophysiology of PD.

## 5. Conclusions

This study demonstrates that patients with PD exhibit a significantly greater PVS burden compared with healthy controls, particularly in the basal ganglia and centrum semiovale. Quantitative measures, especially the largest-per-slice PVS counts, revealed robust group differences with partial right-hemisphere lateralization. Qualitative visual ratings supported these findings, indicating consistent PVS involvement in PD. Although PVS diameters did not differ significantly between groups, the markedly increased number of PVS shows that burden-related metrics may be more sensitive to disease-associated changes.

Importantly, the strong associations between the largest-per-slice PVS burden and lower cognitive performance underscore the potential of PVS quantification as a non-invasive biomarker for cognitive decline in PD. These findings advance current understanding of glymphatic dysfunction and its role in PD neurodegeneration, supporting further longitudinal research to evaluate PVS measures as diagnostic and disease-progression markers.

## Figures and Tables

**Table 1:** Overview of visual rating scales for PVS assessment.

Scoring System	Basis of Rating	Brain Regions Evaluated	Grading Criteria
Potter scale [28]	Number of PVS	Basal ganglia, centrum semiovale, midbrain	0 = none; 1 = <10; 2 = 11–20; 3 = 21–40; 4 = >40; Midbrain 0–1 scale
Heier scale [29]	Diameter of PVS	Basal ganglia	1 = <2 mm; 2 = 2–3 mm; 3 = >3 mm
Adams scale [30]	Number and size of PVS	Basal ganglia, centrum semiovale, hippocampus, midbrain	0–4 scale based on number and size (>1 mm)
Zhu scale [31]	Number of PVS	Basal ganglia, cerebral white matter	For BG: 1 = ≤5, 2 = 6–10, 3 = >10 but countable, 4 = innumerable. For WM: 1 = ≤10 total, 2 = >10 total but <10 in highest section, 3 = 10–20 in highest section, 4 = >20 in highest section
Patankar scale [32]	Number of PVS	Basal ganglia, centrum semiovale, midbrain, subinsular region	For BG: 0 = only one side and <5, 1 => 5 on one side or any in nucleus lentiformis, 2 = any in nucleus caudatus. For CS: 0 = none, 1 = <5 per side, 2 = >5 on one or both sides. Subinsular region: 0 = none, 1= <5 on one side, 2 = >5 on one or both sides

**Table 2:** Demographic characteristics of the study groups. *p*-values were calculated using Mann–Whitney U tests for continuous variables (age, MoCA) due to non-normal distribution and Pearson’s chi-square test for categorical variables (gender).

Variable	Patients (*n* = 21)	Controls (*n* = 21)
Age (years)	66.0 ± 12.2 Median: 64	62.2 ± 7.7 Median: 64
MoCA score	25.60 ± 2.74 Median: 26	28.10 ± 1.82 Median: 29
Gender, (%)	11 male (52.4%) 10 female (47.6%)	10 male (47.6%) 11 female (52.4%)

**Table 3:** Median PVS burden scores assessed by the Potter scale in PD patients and controls across different regions of the brain.

Region	PD Patients (Median)	Controls (Median)	*p*-Value
Right basal ganglia	2.0	1.0	<0.001
Left basal ganglia	2.0	1.0	0.025
Right centrum semiovale	3.0	2.0	<0.001
Left centrum semiovale	3.0	2.0	0.001

## Data Availability

The data presented in this study are available on request from the corresponding author.

## Supplementary Materials

The following supporting information can be downloaded at: https://www.mdpi.com/article/10.3390/medicina62040613/s1.
