# Supplementary material for: Quantitative and Qualitative MRI Assessment of Perivascular Spaces in Parkinson’s Disease Patients
Source: Medicina (Kaunas). 2026 Mar 24;62(4):613. doi: 10.3390/medicina62040613 (PMC13117765; doi:10.3390/medicina62040613)
Supplement: Supplementary file 1 [file medicina-62-00613-s001.zip › medicina-4179332-supplementary.pdf]

Supplementary Materials

Table S1. Group comparisons of PVS metrics including the full dataset without exclusion of the age outlier (32-year-old participant).

Variable	PD patients (mean ± SD)	Controls (mean ± SD)	p-value*
BG total PVS (right)	43.81 ± 20.22	25.31 ± 14.60	<0.001
BG total PVS (left)	40.95 ± 20.68	22.72 ± 11.45	<0.001
CS total PVS (right)	147.62 ± 56.51	75.90 ± 44.31	<0.001
CS total PVS (left)	145.05 ± 58.58	68.00 ± 37.62	<0.001
BG largest-per-slice (right)	13.24 ± 6.77	7.21 ± 2.77	<0.001
BG largest-per-slice (left)	11.57 ± 4.32	8.00 ± 2.75	0.002
CS largest-per-slice (right)	28.76 ± 9.87	16.59 ± 8.65	<0.001
CS largest-per-slice (left)	25.86 ± 8.03	16.21 ± 7.56	<0.001
BG diameter (right)	2.36 ± 0.60	2.10 ± 0.56	0.109
BG diameter (left)	2.10 ± 0.55	2.19 ± 0.83	0.672
CS diameter (right)	2.13 ± 0.74	1.70 ± 0.39	0.010
CS diameter (left)	1.89 ± 0.43	1.69 ± 0.39	0.041

*p-values were calculated using the Mann–Whitney U test.
